# Pedestrian Detection by Novel Axis-Line Representation and Regression Pattern

**DOI:** 10.3390/s21103312

**Published:** 2021-05-11

**Authors:** Mengxue Zhang, Qiong Liu

**Affiliations:** School of Software Engineering, South China University of Technology, Guangzhou 510006, China; 201820137814@mail.scut.edu.cn

**Keywords:** pedestrian detection, object representation, axis line, road scene

## Abstract

The pattern of bounding box representation and regression has long been dominant in CNN-based pedestrian detectors. Despite the method’s success, it cannot accurately represent location, and introduces unnecessary background information, while pedestrian features are mainly located in axis-line areas. Other object representations, such as corner-pairs, are not easy to obtain by regression because the corners are far from the axis-line and are greatly affected by background features. In this paper, we propose a novel detection pattern, named Axis-line Representation and Regression (ALR), for pedestrian detection in road scenes. Specifically, we design a 3-d axis-line representation for pedestrians and use it as the regression target during network training. A line-box transformation method is also proposed to fit the widely used box-annotations. Meanwhile, we explore the influence of deformable convolution base-offset on detection performance and propose a base-offset initialization strategy to further promote the gain brought by ALR. Notably, the proposed ALR pattern can be introduced into both anchor-based and anchor-free frameworks. We validate the effectiveness of ALR on the Caltech-USA and CityPersons datasets. Experimental results show that our approach outperforms the baseline significantly through simple modifications and achieves competitive accuracy with other methods without bells and whistles.

## 1. Introduction

Pedestrian detection is a necessary prerequisite and key component of recent research hotspots (e.g., pedestrian reidentification [1,2,3], human pose estimation [4]), for these tasks it is necessary to detect all the existing pedestrians accurately from images or videos before they go to the next step. In engineering fields, pedestrian detection is also an urgent need in the Advanced Driving Assistance System (ADAS) to help to reduce the occurrence of people-vehicle collisions, or in smart buildings for air conditioner control and monitoring systems [5]. The main purpose of pedestrian detection is to localize and classify each pedestrian accurately in images or videos. Traditional pedestrian detection methods include handcrafted feature descriptors (e.g., HOG [6], Haar [7]) for pedestrians based on basic features such as texture and gradient, which is intuitive but hard to design manually due to the diversity of pedestrian samples. With the rapid development of CNN-based general object detection, many researchers modify the benchmark general object detection method to suit pedestrian detection, such as Adapted FRCNN [8] and RPN+BF [9]. These methods can learn features automatically and achieve significant improvement in pedestrian detection. Generating 4-d rectangular proposals, and computing confidence for each proposal to get the final results, is the most common detection pattern. In recent years, there have been other forms of detection patterns such as CornerNet [10], which detects the left-top and right-bottom points and matches the two corners by post processing to represent an object. Later, CenterNet [11] adds the center-point as extra information to the two corners in CornerNet. 

Compared with the above patterns, we believe detecting the axis-line of a pedestrian is more effective. From the perspective of feature extraction, the internal features of a pedestrian cannot be effectively sensed in the process of detecting corners, because the corners are actually outside the pedestrian and easily influenced by the background information. The axis-line is located at the position with the strongest internal features of a pedestrian, and there is no need to match key points where errors may occur. From the perspective of localization target regression in mainstream detection patterns, the network needs to learn 4-d coordinates (e.g., Faster RCNN [12], MS-CNN [13]), while using our method we only need to learn the 3-d coordinates of the center-point position and the height of the axis-line (or the x-coordinate of the center-point and y-coordinates at both ends of the axis-line), which has the benefits of a smaller hypothesis space and simplifies the localization targets. Besides, for a pedestrian of small-scale, directly learning the axis-line is more feasible compared with accurately learning four edges of a bounding box.

In this work, we named the above pattern as Axis-line Representation and Regression (ALR) and introduced it into both anchor-free and anchor-based methods. For the anchor-free method, RepPoints [14], we designed an ALR pattern that cooperates well with its sampling modules based on deformable convolutions [15] (DCN). To be consistent with the benchmark dataset annotations, we proposed a line-box transformation method by the prior average pedestrian aspect ratio. We found that a different base-offset of the kernel would influence the performance of the detector through different initial receptive fields. Thus, a base-offset initialization strategy was proposed to further improve detection performance by forcing the aspect ratio of the DCN kernel close to the pedestrian aspect ratio. For the anchor-based method Faster R-CNN [12] (simplified as FRCNN), we also introduced the ALR pattern into both the RPN and the detection head. Although the modifications made in the two types of methods are slightly different in implementation, they all follow the main idea of ALR.

Several experiments were carried out on two pedestrian detection benchmark datasets of Caltech-USA [16] and CityPersons [8] to verify the effectiveness of the proposed ALR pattern. Our RPDet+ALR (RPDet means RepPoints detector) obtained absolute gains of 3.6% and 4.6% in MR^−2^ compared to the baseline method on the Caltech-USA test set and CityPersons validation set, respectively. Our FRCNN+ALR achieved an MR^−2^ of 6.5% on the Caltech-USA dataset without any tricks, which is comparable with some state-of-the-art methods. On the CityPersons dataset, FRCNN+ALR also obtained an absolute gain of 1.4% in MR^−2^. The main contributions of this work are summarized as follows:

1 We propose a detection pattern ALR, which uses a simpler 3-d axis-line representation and regression strategy as an alternative to the traditional 4-d bounding box to obtain purer and stronger internal information of pedestrians in road scenes. In addition, we propose a line-box transformation method to fit the benchmark annotations. In particular, the idea of ALR can be introduced into both anchor-free and anchor-based methods.
2 We propose a deformable convolution base-offset initialization strategy towards a more aligned receptive field, and further improvement of detection performance by forcing the aspect ratio of the deformable convolution kernel close to the pedestrian aspect ratio.
3 Several experiments are carried out on two benchmark datasets (the Caltech-USA and the CityPersons) to demonstrate the effectiveness and generalization of the proposed ALR pattern in both anchor-free and anchor-based methods.

The remainder of this paper is organized as follows: Section 2 introduces the related work. Section 3 introduces the proposed ALR pattern and its applications in both the anchor-free method and anchor-based method. Our deformable convolution base-offset initialization strategy and the line-box transformation method are also introduced in Section 3. Section 4 provides the experimental results related to the proposed ALR pattern on two benchmark datasets and verifies effectiveness and generalization by comparisons with the baseline methods and other methods. Section 5 concludes this paper.

## 2. Related Work

### 2.1. Generic Object Detection

In the deep learning era, an object detection task is usually modeled as a problem of classification and regression of candidate regions. In one-stage detectors, these candidate regions are predefined anchors. In two-stage detectors, the candidate regions are proposals generated by the region proposal network (RPN) [12], whose purpose is to classify and regress anchors. Detectors that utilize predefined anchors to cover possible object positions, scales and aspect ratios are called anchor-based detectors [12,13,17,18,19]. They first lay anchors with different scales and aspect ratios on the whole input image, then perform classification and regression based on these prior regions to obtain the final results. The common detection pattern used by anchor-based methods is the 4-d bounding box representation and regression, which is effective but has limitations to the performance and robustness of detectors because it cannot accurately represent the location and will inevitably contain irrelevant information of background or other objects.

In recent years, object detection methods [10,11,20,21,22] tended not to use the predefined anchors but to directly classify and locate objects from each position on the feature maps. Beyond the premise of anchors, methods using various detection patterns have sprung up. CornerNet [10] predicts the top-left and bottom-right corners of the object, enhances the corner features through the corner pooling strategy, and finally performs corner pairing by embedding vectors. CenterNet [11] additionally predicts a center point based on CornerNet to improve localization accuracy. PLN [20] regresses the corner/center points of the bounding box and their links using a fully convolutional network [23]. The above methods attempt to represent objects in a more flexible way, but they may suffer from misdetection or mismatching of corners. ExtremeNet [24] converts object detection into an appearance-based key-point estimation problem. It detects four extreme points and a center point for each object, then groups these key-points by geometric relationships. RepPoints [14] uses a deformable convolution module to automatically learn a set of representative points, which realizes flexible feature sampling and avoids the drawbacks of anchors. We think RepPoints has the potential for pedestrian detection, so we choose it as one of the baseline methods.

### 2.2. Pedestrian Detection

An early pattern of pedestrian detection is using bottom-up representations, such as the deformable part-based model (DPM) [25] and its variants [26,27]. DPM consists of a coarse root template and a set of high-resolution parts templates and their relative position relationships. In each template, HOG is used to extract local features. However, it may suffer from limitations brought by handcrafted features.

The success and popularity of Faster R-CNN [12] motivated the construction of CNN-based pedestrian detection methods. RPN+BF [9] uses RPN followed by boosted forests (BF) as classifiers on shared high-resolution feature maps. Adapted FRCNN [8] uses five simple modifications on the Faster R-CNN and obtains competitive performance for pedestrian detection on the Caltech-USA [16] dataset. ATT-part [28] uses an attention mechanism across channels to represent various occlusion patterns in one single model based on Faster R-CNN. Similar to MS-CNN, SA-FRCNN [29] adopts the divide-and-conquer philosophy that consists of two subnetworks for pedestrians of large-size and small-size, respectively. Bi-Box [30] predicts a visible-part box and full-body box for each pedestrian to produce complementary outputs, as well as a criterion for selecting positive training examples, which contributes largely to heavily occluded pedestrian detection. Double Anchor [31] detects the body and head for each person simultaneously with the help of a crossover strategy and develops a Joint NMS module for robust postprocessing. Like Bi-Box and Double Anchor, there are other methods, e.g., MGAN [32], PedHunter [33] and JointDet [34], to help pedestrian detection by additional detection of the visible part for each pedestrian, which promotes further progress of pedestrian detectors. Most of the above methods still follow the bounding box representation and set it as the regression target as with generic object detection.

With the popularity of anchor-free methods, other representations of pedestrians have emerged. CSP [35] simplifies pedestrian detection into a straightforward center/scale prediction task. Based on CSP, APD [36] introduces four branches to explicitly model the pedestrians’ four semantic attributes, i.e., center, scale, offset and pedestrian-oriented attributes, in a high-level feature detection fashion, as well as an NMS strategy to distinguish pedestrians from highly overlapping groups. TLL+MRF [37] performs pedestrian detection as key-point detections and their associations. It devises an FCN-based network to locate the topological somatic line with a postprocessing scheme based on Markov Random Field (MRF) for multiscale pedestrian detection. In this work, we propose to detect a pedestrian by regressing the axis-line, which is defined by the position of the center point and its height. It is simpler than TLL+MRF and gets rid of the additional computations as well as pairing errors brought by key-point association.

## 3. Proposed Method

To obtain purer and recognizable features, and to simplify network learning at the same time, we propose a detection pattern that uses the axis-line as the object representation and regression target, denoted by ALR. The ALR can be applied to anchor-free and anchor-based methods, so we integrate it into both types of method with slight differences in implementation. In the following, we describe how we introduce the ALR pattern to the anchor-free framework RPDet, and the extra changes we made to help the network perform better. Then we describe how we introduce the ALR pattern to the classic anchor-based framework, Faster R-CNN.

### 3.1. Introducing ALR into the Anchor-Free Method

Following the RPDet [14], we use the center point as the initial representation for each object, then a set of sampling points is determined in two steps. In the first step, the position of sampling points is obtained by learning a set of offsets from the center point, which can be denoted as offset 1. In the second step, the network learns another set of offsets with the help of a deformable convolutional module, denoted by offset 2, then it refines the positions of sampling points based on offset 1. Besides, a parallel branch is constructed to judge whether a set of points expresses a pedestrian or not.

**Figure 1:**
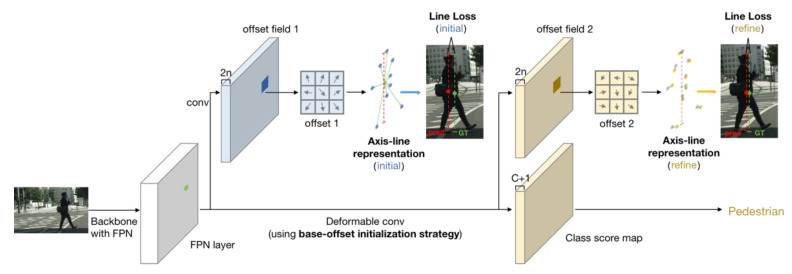
Overview of the proposed RPDet+ALR framework. Different from the original RPDet, we use the axis-line representation in initial/refine stages and the learning is supervised by line loss during training. Besides, the deformable convolution is strengthened by our base-offset initialization strategy. For clear illustration, we only draw the pipeline of one scale of FPN feature maps. All of our modifications are in bold.

To introduce our proposed ALR pattern into the RPDet, we made three main designs: the axis-line representation and regression, the deformable convolutional module with base-offset initialization strategy and a line-box transformation method. The overall architecture of the proposed RPDet+ALR is shown in Figure 1.

#### 3.1.1. Axis-Line Representation and Regression

As previously discussed, the 4-d detection patterns (e.g., CornerNet [10]) cannot accurately locate a pedestrian or eliminate the effects of irrelevant information (e.g., Faster R-CNN [12]). Our ALR pattern only needs to learn three parameters to represent an axis-line located at the internal central area of a pedestrian with pure and semantically significant features, which improves the quality of distinctive features extraction and help the detector to distinguish different pedestrians. The specific implementation is as follows.

Following the RPDet, we first model a set of adaptive sample points in initial and refine step as $R_{i}$ and $R_{r}$:

(1) $$R_{i}={\left\{(x_{k},y_{k})\right\}}_{k=1}^{n}$$

(2) $$R_{r}={\left\{(x_{k}+△x_{k},y_{k}+△y_{k})\right\}}_{k=1}^{n}$$

where *n* is the total number of sample points and it is set to 9 by default.

Define $R_{x}$ and $R_{y}$ as the set of $x_{k}$ and $y_{k}$ values of all *n* elements in a sample point set R, respectively. Then we can define the axis-line l as:

(3) $$l=(x_{center},y_{top},y_{bottom})$$

where

(4) $$x_{center}=\frac{1}{n}\sum_{k=1}^{n}x_{k}\in R_{x}$$

(5) $$y_{top}=\min y_{k}\in R_{y}$$

(6) $$y_{bottom}=\max y_{k}\in R_{y}$$

where in initial and refine steps, R refers to $R_{i}$ and $R_{r}$ respectively. $y_{top}$ equals the minimum of $y_{k}$ because we set the top-left corner of the input image as the origin of the coordinate system, and so does $y_{bottom}$.

In this way, we obtain an axis-line for each pedestrian based on its corresponding adaptive sample point sets. Now the network does not need to explicitly learn *n* sets of offsets for each object, but only needs to learn three parameters related to the axis-line, which simplifies the network learning target. During training, we transform each ground truth (GT) box $G=\left\{\left(x_{tl},y_{tl}\right),\left(x_{br},y_{br}\right)\right\}$ to GT line and denote it by g as:

(7) $$g=(\frac{x_{tl}+x_{br}}{2},y_{tl},y_{br})$$

where ($x_{tl},y_{tl})$ and $\left(x_{br},y_{br}\right)$ are the top-left corner and bottom-right corner of each GT box, respectively.

Then the network is forced to regress the axis-line supervised by loss function $L_{reg}\left(l,g\right)=SmoothL1\left(l-g\right)$, in which ***SmoothL1*** Loss [38] is a widely used robust loss function for regression in object detection networks.

The total training loss is:

(8) $$L(\{p_{i}\},\{l_{i}\},\{l_{r}\})=\frac{\mu_{1}}{N_{cls}}\sum_{j=1}^{N_{cls}}L_{cls}(p_{i}^{j},c^{j})+\frac{\mu_{2}}{N_{p}}\sum_{j=1}^{N_{p}}c^{j}L_{reg\_i}(l_{i}^{j},g^{j})+\frac{\mu_{3}}{N_{p}}\sum_{j=1}^{N_{p}}c^{j}L_{reg\_r}(l_{r}^{j},g^{j})$$

where $L_{cls}$ is the Focal Loss [19] for classification, $L_{reg\_i}$ and $L_{reg\_r}$ are the regression loss functions for the initial step and refine step, respectively, $N_{cls}$ is the total number of sample point sets, $N_{p}$ is the number of positive sample point sets, $p_{i}^{j}$ is the classification confidence of sample *j* during the initial step and $c^{j}$ is the associated GT label of sample *j*. We only compute regression loss for positive sample pointsets by judging whether the GT label $c^{j}=1$ or not. If $c^{j}=1$, sample j is positive, otherwise it is a negative sample. The coefficients $\mu_{1}$, $\mu_{2}$ and $\mu_{3}$ are the weights of different parts of the total loss. In our experiments, $\mu_{1}=1.0$, $\mu_{2}=0.5$ and $\mu_{3}=1.0$ by default. 

#### 3.1.2. Deformable Convolution Base-Offset Initialization Strategy

**Figure 2:**
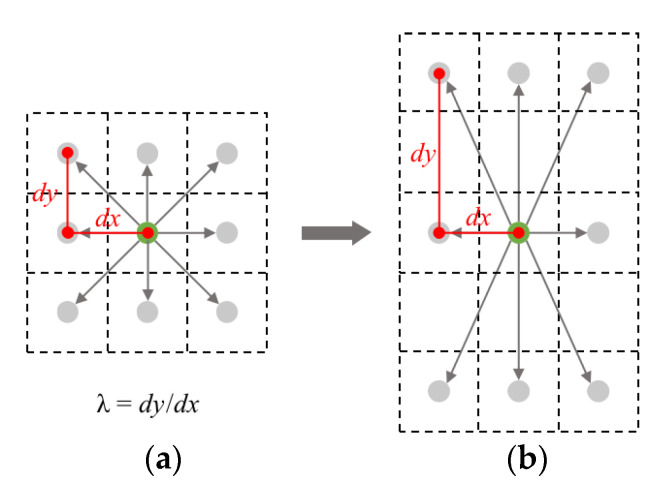
The base-offset of deformable convolution kernel (calculated by λ=dy/dx). (**a**) standard base-offset λ=1; (**b**) base-offset initialization with λ>1.

The receptive field of the standard convolution kernel is square, but its effective receptive is not necessarily square because the object may be deformed, so deformable convolution [15] is proposed to overcome the above limitation. The deformable convolution module adds an offset to each sample point generated from the center point, which achieves random sampling near the current position instead of being limited to the standard regular grid points, and makes the effective receptive field more flexible. As shown in Figure 2, the base-offset λ is 1 in standard deformable convolution. In fact, pedestrians are mostly like slim rectangles in road scenes that are not aligned with the initial shape of deformable convolution. Thus, distances between the initial position and the target position for each sample point of the same kernel may vary a lot, making it difficult for the network to properly update the position of all the sample points in one back-propagation, which increases the difficulty in learning. 

In this work, we propose a deformable convolution initialization strategy to stretch the kernel into a slim rectangle by changing its base-offset λ=dy/dx, where dx = 1, because we only change the base-offset in *y*-direction. In our experiments, we achieved the best detection performance when λ was set to 4.

#### 3.1.3. Line-Box Transformation Method

**Figure 3:**
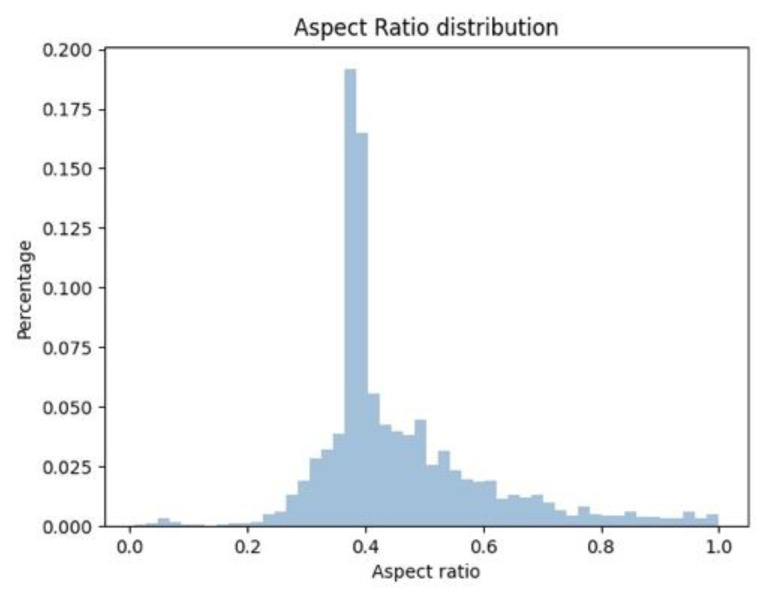
Histogram of pedestrian aspect ratio in the Caltech-USA dataset.

To evaluate the performance of our detector by using the benchmark dataset Caltech-USA [16] and CityPersons [8], we needed to transform the axis-line to a bounding box, consistent with the form of annotations. In this work, we analyzed the proportion of pedestrian samples with different aspect ratios in the Caltech-USA dataset. It can be seen from Figure 3 that the aspect ratio of most pedestrian samples is close to 0.4, which is consistent with the statement in [16] that the average aspect ratio r of pedestrians is close to 0.41. What’s more, the aspect ratio of pedestrian full-body annotations is fixed to 0.41 in the CityPersons dataset. 

Thereby, we can simply transform the line representation to the bounding box representation BBox as:

(9) $$BBox=(x_{center}-\frac{w}{2},y_{top},w,|y_{top}-y_{bottom}|)$$

where

(10) $$w=r\cdot h=r\cdot |y_{top}-y_{bottom}|$$

### 3.2. Introducing ALR into the Anchor-Based Method

For the anchor-based method, we chose Faster R-CNN [12] as our baseline. In this work, we introduce the proposed ALR pattern to Faster R-CNN by replacing the bounding box regression in both the RPN and detection head with the axis-line encoding/decoding method, as well as its corresponding loss calculation manner.

**Figure 4:**
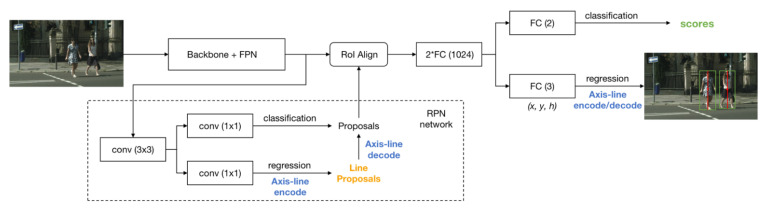
The overall architecture of the proposed FRCNN+ALR.

Figure 4 shows the overall architecture of our FRCNN+ALR. In the subnetwork RPN, a series of anchors are laid out and classified to determine whether they belong to the foreground or the background. Another branch is used to adjust the location and scale of these foreground anchors through axis-line regression to form line proposals, which are transformed to box-shape proposals by axis-line decoder. Then, the network uses RoI Align [39] to obtain fixed-size proposal feature maps from proposals based on the shared feature map. Finally, the detection head determines the specific category of each proposal and performs the axis-line regression again towards a more accurate location and scale of the object.

#### 3.2.1. Axis-Line Encoder and Decoder

In our FRCNN+ALR framework, an axis-line is determined by its center point and height, represented as $l=\left(x_{center},y_{center},h\right)$. To encode the axis-line for regression, we must learn a set of offsets between a predicted axis-line and the associated GT axis-line $l^{*}=\left(x^{*},y^{*},h^{*}\right)$. Following [12], we parameterize the offset set *t* in terms of three dimensions $t_{x}$, $t_{y}$ and $t_{h}$, defined as Equation (11). $t_{x}$ and $t_{y}$ specify the offsets from the center of the predicted axis-line, while $t_{h}$ specifies the log-space offset from the height of the predicted axis-line. Similarly, we can parameterize the offset set from the axis-line of an anchor to the axis-line of its associated GT by $t^{*}$ defined in Equation (12). During training, our goal is to minimize the difference between *t* and $t^{*}$.

(11) $$t_{x}=(x-x_{a})/w_{a},t_{y}=(y-y_{a})/h_{a},t_{h}=\log(h/h_{a})$$

(12) $$t_{x}^{*}=(x^{*}-x_{a})/w_{a},\;t_{y}^{*}=(y^{*}-y_{a})/h_{a},\;t_{h}^{*}=\log(h^{*}/h_{a})$$

where *x*, *y* and *h* denote the predicted center coordinates and height of the axis-line. Variable $x_{a}$, $y_{a}$ and $h_{a}$ are for the anchors’ axis-lines. Variables $x^{*}$, $y^{*}$ and $h^{*}$ are for the GTs’ axis-lines.

To decode the axis-line back to the bounding box for feature extraction and evaluation on the benchmark datasets, we base on the fact that the average aspect ratio r of the pedestrian is about 0.41 (note that r=w/h). We define the bounding box as $bbox=\left(x_{b},\;y_{b},w_{b},h_{b}\right)$ by its center point position, width and height. We can infer bbox through the inverse computation of the offset set *t*, with Equations as follows:

(13) $$x_{b}=x_{a}+r\cdot h_{a}\cdot t_{x},\;y_{b}=y_{a}+h_{a}\cdot t_{y}$$

(14) $$w_{b}=r\cdot h_{a}\cdot \exp(t_{h}),\;h_{b}=h_{a}\cdot \exp(t_{h})$$

#### 3.2.2. Loss Calculation Manner

With the above definitions, our total training loss is defined as follows:

(15) $$L(\{s_{i}\},\{t_{i}\})=\frac{\alpha }{N_{cls}}\sum_{i=1}^{N_{cls}}L_{cls}(s_{i},c_{i}^{*})+\frac{\beta }{N_{p}}\sum_{i=1}^{N_{p}}c_{i}^{*}L_{reg}(t_{i},t_{i}^{*})<\mathrm{obj}/>$$

where i is the index of samples and $N_{cls}$ is the total number of samples. $N_{p}$ is the number of positive samples, $s_{i}$ is the predicted confidence of sample i being a pedestrian and $c_{i}^{*}$ is the associated GT label of sample *i*. Notably, we only compute regression loss for positive samples by judging whether the GT label $c_{i}^{*}=1$ or not. If $c_{i}^{*}=1$, sample i is positive, otherwise it is a negative sample. $t_{i}$ is a 3-d vector representing the parameterized coordinates of the predicted axis-line, and $t_{i}^{*}$ is that of the GT. The classification loss $L_{cls}$ is the cross-entropy loss over two classes (pedestrian vs. not pedestrian). For the regression loss $L_{reg}$, we use the L1 loss function. α and β are the balanced weights for $L_{reg}$. In our experiments, α and β are set to 1 by default.

## 4. Experiments

We evaluate our approach on two pedestrian detection benchmark datasets: Caltech-USA [16] and CityPersons [8].

### 4.1. Datasets and Evaluation Metric

#### 4.1.1. Caltech-USA Dataset

The Caltech-USA pedestrian dataset includes 250,000 frames with a total of 350,000 extensively labeled bounding boxes around 2300 unique pedestrians. It groups pedestrians by their height in pixels into three scales: near (80 or more pixels), medium (between 30–80 pixels), and far (30 pixels or less). Occluded pedestrians are annotated with two bounding boxes that denote the visible and full pedestrian extent respectively, then subdivided into bare (no occlusion), partial occlusion (1–35% area occluded) and heavy occlusion (35–80% occluded). The fraction of occlusion is computed as one minus the visible pedestrian area divided by the full pedestrian area. 

The Reasonable subset is the most widely used subset for evaluating pedestrian detection approaches, and includes pedestrians over 50 pixels under no or partial occlusion. For network training, we used the Caltech 10× training set [40] as commonly done in [8,9,28,30,37,41], which provides annotations of higher quality compared to the original annotations. We tested and evaluated our models in the standard testing set using new annotations provided by [40].

#### 4.1.2. CityPersons Dataset

The CityPersons dataset includes about 20,000 identical pedestrian, and over 20% of pedestrian samples overlap with another pedestrian whose IoU is above 0.3. For subset division, it sets up the Reasonable subset by including pedestrians with heights of 50 or more pixels and occlusion ratios between 0% and 35%. The small, middle and large subsets include pedestrians with heights of [50, 75), [75, 100) and [100, ∞) respectively. The partial subset and heavy subset include pedestrians with occlusion ratio in interval [0.1, 0.65) and [0.65, 1), respectively. 

For fair comparison, we trained the models on the standard training set with 2975 images and tested on the validation set with 500 images as commonly done in other works such as [8,28,37].

#### 4.1.3. Evaluation Metric

In our experiments we used the standard log-average miss rate (MR) official evaluation metric of the Caltech-USA and CityPersons datasets. The MR^−2^ is averaged over the false positive per image (FPPI) between 10^−2^ and 10^2^ in log space. Lower is better.

### 4.2. Implementation Details

We implemented the proposed method in MMDetection (OpenMMLab detection toolbox and benchmark). We adopted ResNet-50 pretrained on the ImageNet [42] dataset as the backbone and a five-layer FPN as the neck in both RPDet+ALR and FRCNN+ALR. For experiments based on FRCNN, the initial anchor ratio was set to 2.44, and RoI Align was also used for better feature extraction. We optimized both FRCNN-based and RPDet-based detectors using Stochastic Gradient Descent (SGD) with 0.9 momentum and 0.0001 weight decay on Caltech-USA and CityPersons datasets. Specifically, for the Caltech-USA dataset, a mini-batch contained 16 images with 8 GPU (GTX 1080Ti). The initial learning rate was 0.02 for both RPDet-based and FRCNN-based experiments. For the CityPersons dataset, a mini-batch contained four images with four GPU, the learning rate was set as 0.002 and 0.02 for RPDet-based and FRCNN-based experiments, respectively. Unless otherwise specified, we divided the learning rate by 10 at 8 and 11 epochs with a total of 12 epochs.

### 4.3. Detection Results of RPDet-Based Models

#### 4.3.1. Overall Performance

**Figure 5:**
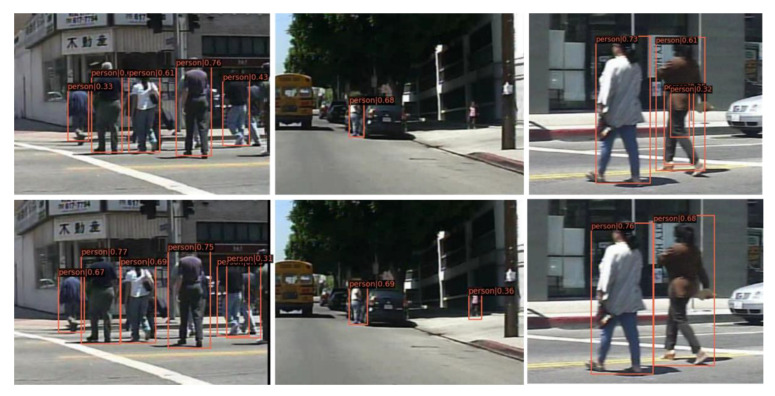
Qualitative results from the baseline method (**the first row**) and our RPDet+ALR (**the second row**) in the Caltech-USA testing set.

To verify the effectiveness of the proposed ALR pattern, we compared the proposed RPDet+ALR with the vanilla RPDet (baseline) on the Caltech-USA and CityPersons datasets. Table 1 shows the detection results on the Caltech-USA dataset. ‘+T’ means using the line-box transformation method, ‘+A’ means using the axis-line representation and regression, ‘+λ’ means using the deformable convolution base-offset initialization strategy. The base-offset λ was set to 4 here for best performance, which will be explained in Section 4.3.3. The scores are log average miss rate (MR^−2^), and all models were trained and tested using new annotations. We can see that the RPDet+ALR outperformed the baseline method RPDet in MR^−2^ by 3.6%, 2.5%, 2.9%, and 2.7% in the Reasonable, all, medium, and heavy subsets, respectively. Qualitative results are shown in Figure 5, where we can see that our method was able to detect occluded or missed pedestrians, and reduced false positives compared to the baseline method.

**Figure 6:**
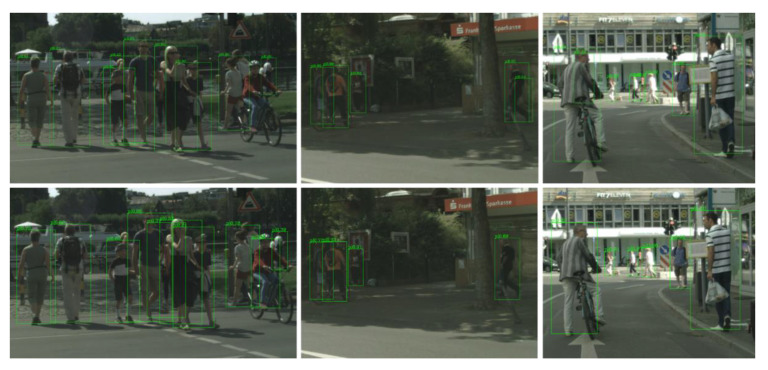
Qualitative results from the baseline method (**the first row**) and our RPDet+ALR (**the second row**) in the CityPersons validation set.

Table 2 shows the detailed results across all the subsets of the CityPersons dataset. Compared with the baseline method RPDet, introducing our ALR pattern led to a significant improvement in each subset. Notably, it improved the MR^−2^ by 4.6% on the Reasonable subset and achieved a huge boost of 7.9%/4.3% on the challenging small and heavy subsets, respectively. The above results demonstrate the effectiveness of the proposed ALR pedestrian detection pattern and show its potential of improving the detector’s ability to handle small-size and occluded pedestrians to some extent. Qualitative results are shown in Figure 6, where we can see that our method achieved more accurate detection and overcame some false positives compared to the baseline method.

#### 4.3.2. Ablation Study

As described in Section 3, we made three improvements to the original RPDet. Here, we performed ablation experiments on the Caltech-USA dataset. The results are shown in Table 2. As we can see, introducing ‘T’ without ‘A’ could still improve the MR^−2^ by 0.8%, which indicates that ‘T’ is also applicable to the original RPDet and its effect is better than the original transformation method ‘moment’. Specifically, it is more proper to find the mean value of each point’s coordinate in the *x*-direction and the minimum/maximum in the *y*-direction, then infer the scale and position of the bounding box according to the pedestrian aspect ratio prior, because it increases fault tolerance during the regression process of each sample point. Since ‘T’ is the prerequisite for ‘A’ to work, we did not conduct the experiment that only used ‘A’. Besides, we can see that introducing ‘T’ and ‘A’ together improved the MR^−2^ by 2.8%, which verifies the effectiveness of our idea of predicting the axis-line for pedestrians. Besides, ‘+λ’ also led to a gain of 1.1% in MR^−2^ on the Reasonable subset, which was set to 4 according to the following exploration results in Table 3. Finally, we introduced ‘T’, ‘A’, and ‘λ’ into the baseline at the same time and reported the best results with an improvement of 3.6% in MR^−2^ on the Reasonable subset.

#### 4.3.3. Influence of Deformable Convolution Base-Offset

To explore the influence of the deformable convolution base-offset on network performance, we changed the base-offset λ from 1 to 6 in the RPDet+ALR framework. It is worth noting that the base-offset here refers to its *y*-component. Intuitively, it is like stretching the square kernel (λ=1) longitudinally. Table 3 shows the detailed results across subsets of the Caltech-USA dataset using new annotations, where K-ratio means the aspect ratio of the deformable convolution kernel. It can be observed that when the value of λ was increased from 1 to 4, the MR^−2^ on the Reasonable subset gradually decreased to 9.4% and achieved the best result. 

However, the MR^−2^ rose to 11.7% and 12.5% when λ increased to 5 and 6, respectively. It indicates that we may find the optimum when the K-ratio falls into the interval [0.33, 0.42], and the extreme K-ratios (e.g., 1.0, 0.27, and 0.23) bring down the detection performance. We think the reason is that the K-ratio is close to the average pedestrian aspect ratio (0.41) when it falls into the interval (0.33, 0.42), so the initial shape of the receptive field is more aligned with the pedestrians, which helps to obtain better performance. Thereby, to further improve the detection accuracy, we set the base-offset of deformable convolutions to 4 in our initialization strategy according to the best results in Table 3 currently.

### 4.4. Detection Results of FRCNN-Based Models

#### 4.4.1. Overall Performance

**Figure 7:**
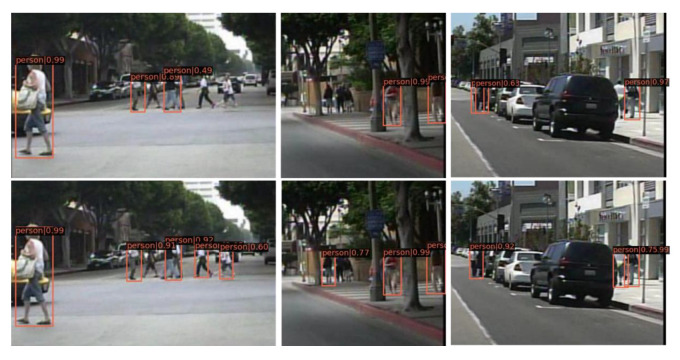
Qualitative results from the baseline method (**the first row**) and our FRCNN+ALR (**the second row**) in the Caltech-USA testing set.

For the anchor-based method, we choose the Faster RCNN [12] as our baseline method, which is denoted as FRCNN in the following. The results of FRCNN+ALR on the Caltech-USA dataset are shown in Table 4. We can see that it outperformed the baseline across all the subsets, i.e., improvement of 2.1% MR^−2^ on the Reasonable subset, 2.5% MR^−2^ on the bare subset, and particularly, 8.1%/5.9% MR^−2^ on the challenging partial/heavy subsets respectively, which indicates that the proposed ALR was able to improve performance of the detector toward better robustness in occlusion cases. Qualitative results are shown in Figure 7, which shows that our FRCNN+ALR was more robust than the baseline method.

**Figure 8:**
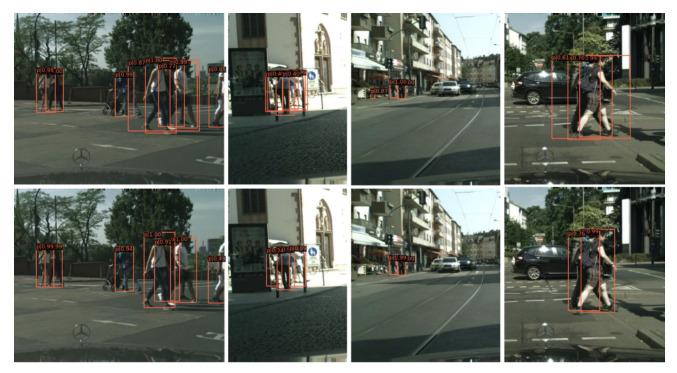
Qualitative results from the baseline method (**the first row**) and our FRCNN+ALR (**the second row**) in the CityPersons validation set.

We also evaluated the proposed FRCNN+ALR on the validation set of the CityPersons dataset. As shown in Table 5, the proposed ALR pattern led to a gain of 1.4% in MR^−2^ on the basis of FRCNN. Besides, it is worth noting that the ALR pattern improved the MR^−2^ by 4.1% on the small subset, which demonstrates that ALR can help the FRCNN to deal with pedestrians of small-scale much better, because pedestrians of small-scale have a lower resolution, making it is easy for the detector to make mistakes. While in the proposed ALR pattern, we first learn the axis-line of a pedestrian to obtain its height in pixels, then infer its width based on the uniform pedestrian aspect ratio prior. This alleviates the negative influence of low resolution because we no longer need to learn the precise 4-d coordinates of the bounding box. Qualitative results are shown in Figure 8, where there are no redundant detection boxes of abnormal shape, whereas they appear in the results of the baseline method. This is due to the fact that we convert the axis-lines of pedestrians into bounding boxes according to a uniform aspect ratio.

#### 4.4.2. Comparison with Other Methods

We compared the proposed FRCNN+ALR pedestrian detection framework with some representative methods on the Caltech-USA testing set, including DeepParts [43], MS-CNN [13], CompACT-Deep [44], ATT-part [28], F-DNN+SS [45], SA-FRCNN [29], RPN+BF [9] and Repulsion Loss [46]. The results are shown in Table 6. It can be seen that the proposed FRCNN+ALR achieved an MR^−2^ of 6.5% on the Reasonable subset, which proves that the ALR pattern and FRCNN are well adapted to each other and comparable to, or even better, than some listed methods. However, the result of the FRCNN+ALR had a gap (2.5% in MR^−2^) with the Repulsion Loss method on the Reasonable subset. One possible reason is that the backbone used in Repulsion Loss was ResNet-101, which was deeper and stronger than our backbone ResNet-50. Another reason is that it used the CityPersons dataset as the pretraining data source with much bigger input images (i.e., ×1.5 scale), which helped to further boost the detection performance, while our FRCNN+ALR framework was directly trained on the Caltech-USA dataset from the beginning without any pretraining. In terms of space complexity, the proposed method had a slight decrease compared with the baseline method because it learned a 3-d axis-line as an alternative to a 4-d bounding box. In terms of inference speed, the baseline method (14.4 fps) and the proposed method (14.1 fps) showed similar results with one GPU.

As shown in Table 7, we also compared the FRCNN+ALR with several state-of-the-art methods on the validation set of the CityPersons dataset, including ATT+vbb [28], Adapted FRCNN [8], TLL+MRF [37], Repulsion Loss [46] and OR-CNN [47]. The results show that our FRCNN+ALR improved the MR^−2^ to 12.5% on the Reasonable subset by introducing simple modification into the FRCNN, which outperformed other methods without any tricks. Notably, the TLL-MRF aimed to detect the topological line of pedestrians by predicting key points of the human body and linking them up with MRF, which is somewhat similar to our idea of detecting the axis-line for each pedestrian. However, our ALR pattern is totally different from TLL in object representation, feature extraction, regression strategy and implementations. Actually, our method is simpler than TLL+MRF because we do not need complicated postprocessing as key points matching by MRF. 

## 5. Conclusions

In this paper, we propose a detection pattern called ALR for promoting pedestrian detection in road scenes. To achieve this, we designed an axis-line representation for the pedestrian and set it as the regression target of the network. We also introduced a line-box transformation method to keep consistency with annotations of the datasets for evaluation. Besides, a base offset initialization strategy is proposed for the deformable convolutional to get a better receptive field and more balanced learning process, which can further promote detection accuracy. Our ALR pedestrian detection pattern is demonstrated to be capable of improving performance for both anchor-based and anchor-free methods on the Caltech-USA and CityPersons datasets.

## Figures and Tables

**Table 1:** Results of RPDet-based models on the Caltech-USA testing set (MR^−2^/%).

Method	+T	+A	+λ	Reasonable	All	Near	Medium	Bare	Partial	Heavy
RPDet				13.0	65.7	2.2	47.7	10.3	34.4	59.1
	√			12.2	64.6	4.6	47.6	10.0	34.9	57.3
			√	11.9	64.0	4.3	46.4	9.9	41.5	59.4
	√	√		10.2	64.9	3.7	48.1	8.7	35.7	56.5
RPDet+ALR (ours)	√	√	√	9.4 (+3.6)	63.2 (+2.5)	1.8 (+0.4)	44.8 (+2.9)	7.1 (+3.2)	33.9 (+0.5)	56.4 (+2.7)

**Table 2:** Comparison of RPDet+ALR with baseline across each subset of the CityPersons validation set (MR^−2^/%).

Method	Reasonable	All	Large	Middle	Small	Bare	Partial	Heavy
RPDet	22.1	53.2	13.2	13.8	32.6	13.6	24.8	73.8
RPDet+ALR (ours)	17.5 (+4.6)	49.7 (+3.5)	10.9 (+2.3)	7.5 (+6.3)	24.7 (+7.9)	9.3 (+4.3)	18.1 (+6.7)	69.5 (+4.3)

**Table 3:** Results of RPDet+ALR with varying λ on the Caltech-USA testing test (MR^−2^/%).

Method	λ	K-Ratio	Reasonable	All	Near	Medium	Bare	Partial	Heavy
RPDet+ALR (ours)	1	1.0	10.2	64.9	3.7	48.1	8.7	35.7	56.5
	2	0.6	9.8	64.6	2.8	48.0	8.2	38.4	58.3
	3	0.42	9.7	64.3	2.1	47.1	7.9	37.8	57.3
	4	0.33	9.4	63.2	1.8	44.8	7.1	33.9	56.4
	5	0.27	11.7	65.0	3.9	48.9	10.4	33.9	58.3
	6	0.23	12.5	65.8	3.7	49.6	10.6	36.8	61.8

**Table 4:** Comparison of FRCNN+ALR with baseline across each subset of the Caltech-USA test set (MR^−2^/%).

Method	Reasonable	All	Near	Medium	Bare	Partial	Heavy
FRCNN	8.6	63.9	1.9	47.2	8.2	28.1	62.1
FRCNN+ALR (ours)	6.5 (+2.1)	62.4 (+1.5)	1.8 (+0.1)	46.1 (+1.1)	5.7 (+2.5)	20.0 (+8.1)	56.2 (+5.9)

**Table 5:** Comparison of FRCNN+ALR with baseline across each subset of the CityPersons validation set (MR^−2^/%).

Method	Reasonable	All	Large	Middle	Small	Bare	Partial	Heavy
FRCNN	13.9	47.0	8.4	6.4	23.6	7.8	14.7	67.5
FRCNN+ALR (ours)	12.5 (+1.4)	46.1 (+0.9)	7.5 (+0.9)	7.5 (−1.1)	19.5 (+4.1)	7.1 (+0.7)	13.0 (+1.7)	67.1 (+0.4)

**Table 6:** Comparisons of the proposed FRCNN+ALR framework with other methods on the standard test set of the Caltech-USA dataset using new annotations (MR^−2^/%).

Method	Backbone	Reasonable
DeepParts	AlexNet	12.9
MS-CNN	VGG16	9.5
CompACT-Deep	VGG16	9.2
FRCNN (baseline)	ResNet50	8.6
ATT-part	VGG16	8.1
F-DNN+SS	VGG16	7.6
SA-FRCNN	VGG16	7.5
RPN+BF	VGG16	7.3
Repulsion Loss	ResNet101	4.0
FRCNN+ALR (ours)	ResNet50	6.5

**Table 7:** Comparisons of the proposed FRCNN+ALR framework with other methods on the validation set of the CityPersons dataset (MR^−2^/%).

Method	Backbone	Reasonable
ATT-vbb	VGG16	16.4
Adapted FRCNN	VGG16	15.4
TLL+MRF	ResNet50	14.4
FRCNN (baseline)	ResNet50	13.9
Repulsion Loss	ResNet101	13.7
OR-CNN	VGG16	12.8
FRCNN+ALR (ours)	ResNet50	12.5

## Data Availability

The data presented in this study are available on request from the author. The data are not publicly available due to involve a certain degree of privacy.
